# Association between body mass index and physical fitness among multiethnic children aged 9–12 years in Zhejiang Province, China: a cross-sectional quantile regression analysis

**DOI:** 10.3389/fpubh.2026.1821894

**Published:** 2026-06-17

**Authors:** Changliang Han, Shipeng Ding, Yuan Feng, Jingtao Wu

**Affiliations:** 1 School of Teacher Education, Lishui University, Lishui, China; 2 College of Physical Education, Dalian University, Dalian, China; 3 School of Physical Education, Leshan Normal University, Leshan, China

**Keywords:** body mass index, children, ethnicity, physical fitness, quantile regression, school health

## Abstract

**Background:**

Evidence on the association between body mass index (BMI) and physical fitness among children of different ethnicities remains limited. Based on a multiethnic sample of students aged 9–12 years in Zhejiang Province, this study examined the association between BMI and physical fitness across different performance levels using quantile regression.

**Methods:**

From September to October 2024, students in grades 3–6 were recruited from primary schools in Zhejiang Province using school-based cluster sampling. Schools were the primary sampling units, and eligible students aged 9–12 years from the four predefined ethnic groups were invited. Physical fitness indicators included lung capacity, 50-meter sprint, sit-and-reach, 1-min rope skipping, 1-min sit-ups, and 50 × 8-meter shuttle run. Quantile regression models assessed BMI–fitness associations across quantiles, adjusting for gender, grade, and ethnicity. Because the analyses were conducted on the final analytic sample without full design-based variance adjustment, the findings should be interpreted as sample-based associations.

**Results:**

Of the 12,000 students approached, 11,560 students with complete data were included in the final analytic sample. The associations between BMI and physical fitness outcomes showed clear quantile-specific patterns. For lung capacity, BMI was positively associated with the outcome across all prespecified quantiles from 0.10 to 0.90. For 50-meter sprint time and 50 × 8-meter shuttle run time, BMI was positively associated with completion time across all prespecified quantiles, indicating that students with higher BMI tended to have poorer speed and shuttle-run performance. The association between BMI and 50-meter sprint time was strongest around the middle quantiles. For sit-and-reach performance, significant positive associations were observed at selected quantiles, including higher quantiles. For 1-min sit-ups and 1-min rope skipping, the direction and strength of the associations varied across quantiles, demonstrating heterogeneous BMI–fitness associations across the distribution of physical performance.

**Conclusion:**

BMI showed quantile-specific associations with multiple physical fitness indicators. Higher BMI was consistently associated with greater lung capacity and longer completion times in the 50-meter sprint and 50 × 8-meter shuttle run, indicating poorer speed-related performance. Associations with sit-ups and rope skipping varied in direction across quantiles, while the positive association with sit-and-reach performance was more evident at selected higher quantiles.

## 1 Introduction

Late childhood and early puberty (approximately ages 9–12) represent a critical period for physical growth and long-term health. Physical fitness during this stage is both an indicator of current health and a predictor of future quality of life and chronic disease risk (1). Accordingly, physical fitness in school-aged children has received increasing research attention worldwide (2). Body mass index (BMI), a widely used indicator of weight relative to height, is commonly applied to assess nutritional status and related health risks (3). Although many studies have examined the relationship between BMI and physical fitness, uncertainty remains regarding how different BMI ranges affect fitness and whether these associations vary across populations (4, 5).

Existing evidence suggests that cultural, lifestyle, and environmental factors may modify the BMI–fitness relationship. Studies in Western populations, especially among European school-aged children, have reported nonlinear associations between BMI and strength, flexibility, and cardiorespiratory fitness (6). In China, previous studies have suggested that physical activity barriers, dietary habits, and regional living environments may shape children’s BMI and physical fitness (7, 8). However, systematic evidence on the relationship between BMI and physical fitness among children of different ethnicities in China remains limited.

Quantile regression is particularly useful for examining relationships across different parts of an outcome distribution (9, 10). Compared with conventional linear regression, it can detect heterogeneous associations between BMI and physical fitness at different fitness levels, thereby supporting more targeted interventions (11). Although other methods may also be used to assess the overall functional form of potential nonlinearity, quantile regression is especially suitable when the research focus is on variation in associations across outcome quantiles. Previous studies have shown that BMI–fitness associations may differ across quantiles, with positive associations at lower quantiles and reversed patterns at higher ranges (12).

Nevertheless, current research has largely focused on average effects and has paid less attention to variation across fitness quantiles (6). In addition, the roles of cultural background, lifestyle, and local environment in shaping children’s BMI–fitness associations remain insufficiently understood (7, 13). Zhejiang Province, with its ethnic diversity and regional variability, provides a useful setting for examining these issues. Therefore, rather than assuming fixed differences between specific ethnic groups, the present study included ethnicity as a covariate and explored whether BMI–fitness associations varied across the physical fitness distribution.

Given these gaps in the literature and the ethnic diversity of Zhejiang Province, this study employed quantile regression to examine the associations between BMI and physical fitness among multiethnic students aged 9–12 years in Zhejiang Province, with particular attention to variation across outcome quantiles.

## 2 Methods

### 2.1 Participants and recruitment

The EUROFIT (14) and Presidential Youth Fitness Program (PYFP) (15) protocols were consulted as international references for standardized youth fitness assessment; however, the complete EUROFIT or PYFP test batteries were not administered in this study. The selected indicators were those included in, or compatible with, the Chinese school-based physical fitness assessment system.

Between September and October 2024, a school-based cluster sampling approach was used across multiple primary schools in Zhejiang Province. Schools were treated as the primary sampling units. Within selected schools, students in grades 3–6 who met the inclusion criteria were invited to participate according to the field survey plan. The initial recruitment target of approximately 12,000 students was determined according to the field survey capacity of the participating schools and research team, the need to ensure adequate representation of key subgroups defined by ethnicity, gender, and grade, and the anticipated exclusion of some records during data cleaning because of missing key variables or extreme outliers.

Eligible participants were children aged 9–12 years who belonged to one of the four target ethnic groups, namely Han, She, Manchu, or Tujia, had long-term residence in Zhejiang Province, and provided guardian consent. Exclusion criteria included chronic conditions affecting physical development, physical disabilities potentially influencing measurements, and inability or unwillingness to complete the physical fitness tests.

Data were collected by trained physical education teachers and researchers in standardized school-based testing settings. Detailed assessment methods and research procedures are described below.

### 2.2 Ethical considerations

This study was approved by the Academic Ethics Committee of Leshan Normal University (Approval No. LSNU20240916) and conducted in accordance with the ethical principles of the Declaration of Helsinki. Before data collection, detailed study information was provided to participants and their guardians, and written informed consent was obtained from guardians with verbal assent from students. During data collection and data processing, personal identifiers were replaced with unique codes. All data were anonymized and entered using a dual-check procedure, and access to identifiable information was restricted to the core research team.

### 2.3 Assessment methods

The assessment framework was based mainly on the National Student Physical Health Standard (2014 Revision) (16), which is the official school-based physical fitness assessment system used in China. International youth fitness protocols, including EUROFIT (14) and PYFP (15), were used only as methodological references for standardized testing principles. Therefore, the present study should not be interpreted as applying the full EUROFIT or PYFP batteries. The framework examined BMI and six school-based physical fitness indicators among primary school students aged 9–12 years.

Morphological measurements included height and weight, and BMI was calculated as weight in kilograms divided by height in meters squared. BMI status was then categorized as underweight, normal weight, overweight, or obese according to the age- and sex-specific classification criteria for Chinese school-aged children. The corresponding cut-off values were applied separately for each age and sex group during data processing.

The physical fitness evaluation encompassed six indicators: lung capacity measured using an electronic spirometer, 50-meter sprint time measured in seconds from standing start to chest crossing the finish line, sit-and-reach performance measured as the maximum distance in centimeters from two attempts on a specialized bench with feet flat against the testing board, 1-min sit-ups counted as valid repetitions with hands crossed on chest and knees bent, 1-min rope skipping counted as continuous jumps, and 50 × 8-meter shuttle run time measured as the total time in seconds for eight round trips. All tests were administered by trained researchers and physical education teachers using calibrated equipment. Medical personnel were present at each site, and measurements were recorded independently by two staff members.

### 2.4 Research procedures

Data collection was conducted at participating schools from September to October 2024. Before testing, schools, students, and guardians were informed about the study procedures. Written informed consent was obtained from guardians, and verbal assent was obtained from students. Testing was carried out in designated indoor or outdoor areas using calibrated equipment and standardized procedures under the supervision of trained staff. Dual recording was used for data entry, first-aid support was available on site, and all records were anonymized using unique identifiers after data collection, with access to identifiable information restricted to the core research team.

### 2.5 Statistical analysis

The data analysis was performed using SPSS 27.0 and R 4.2.0 statistical software on the cleaned dataset. Initial descriptive statistics characterized the study population by calculating means, standard deviations, percentiles, and frequency distributions for all demographic and physical fitness variables (17). Group comparisons by gender, grade, and ethnicity employed independent t-tests and one-way analysis of variance (ANOVA) (18), with non-parametric alternatives (Mann–Whitney U or Kruskal-Wallis tests) used when normality assumptions were violated (19).

The primary analysis employed quantile regression (20) to assess the association between BMI and each physical fitness outcome across different segments of the conditional outcome distribution. Gender, grade, and ethnicity were included as covariates in all models. Six physical fitness outcomes were analyzed separately: lung.

capacity, 50-meter sprint time, sit-and-reach, 1-min sit-ups, 1-min rope skipping, and 50 × 8-meter shuttle run time. Models were fitted at prespecified quantiles from 0.10 to 0.90 in increments of 0.10 (21), with the aim of identifying quantile-specific heterogeneity rather than relying only on mean-based estimates. For transparency and consistency with the graphical presentation, estimates for all prespecified quantiles were reported regardless of statistical significance. Because the analyses were conducted on the final analytic sample and did not fully incorporate design-based variance estimation for the school-based cluster sampling design, the estimates are interpreted as sample-based associations.

## 3 Results

### 3.1 Demographic characteristics

Of the 12,000 students initially approached, 11,560 had complete data and were included in the final analytic dataset after excluding records with missing key variables or extreme outliers. Table 1 summarizes the baseline characteristics of these 11,560 participants. The sample included students from grades 3–6, both genders, and four ethnic groups, with Han students forming the largest subgroup. Overall, the gender distribution was relatively balanced, and most participants achieved pass, good, or excellent classifications in the physical fitness assessment. Detailed frequencies and percentages are presented in Table 1.

**Table 1:** Baseline characteristics and physical fitness classification of the study sample.

Name	Options	Frequency	Percentage (%)	Cumulative percentage (%)
Grade	Grade 3	2,912	25.19	25.19
	Grade 4	2,712	23.46	48.65
	Grade 5	2,776	24.01	72.66
	Grade 6	3,160	27.34	100.00
Ethnicity	Han	6,828	59.07	59.07
	She	2,758	23.86	82.93
	Manchu	1,068	9.24	92.17
	Tujia	906	7.84	100.00
Gender	Male	6,080	52.60	52.60
	Female	5,480	47.40	100.00
Total score classification	Excellent	4,144	35.85	35.85
	Good	3,696	31.97	67.82
	Pass	3,528	30.52	98.34
	Fail	192	1.66	100.00
Total		11,560	100.00	100.00

Percentages are column percentages. Grade = school grade at testing (3–6). Ethnicity = self-reported and cross-checked with school rosters (Han, She, Manchu, Tujia). Physical fitness total score classification follows the National Student Physical Health Standard (2014 Revision) (Excellent, Good, Pass, Fail). No missing data remained in the final analytic dataset after data cleaning. Data cleaning included checking data completeness and consistency and excluding records with missing key variables or extreme outliers before analysis.

### 3.2 Quantile regression analysis of the association between BMI and physical fitness outcomes

**Figure 1:**
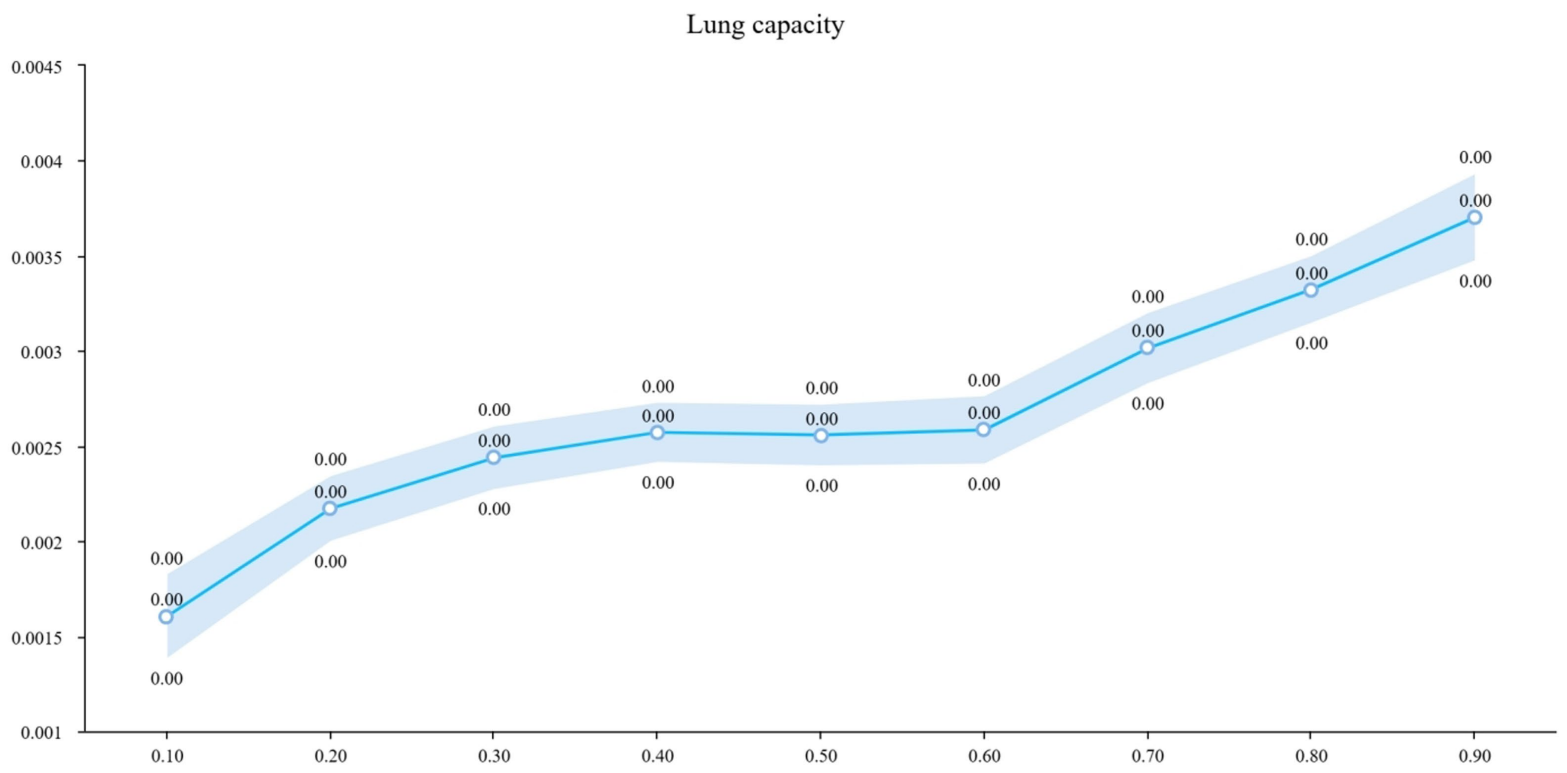
Quantile regression results for BMI and lung capacity. This figure shows the quantile-specific association between BMI (kg/m^2^) and lung capacity (mL). The y-axis represents the BMI regression coefficient (*β*) for lung capacity, and the x-axis represents the quantile (*τ*) from 0.10 to 0.90. The shaded area represents the 95% confidence interval. Models were adjusted for gender, grade, and ethnicity.

**Figure 2:**
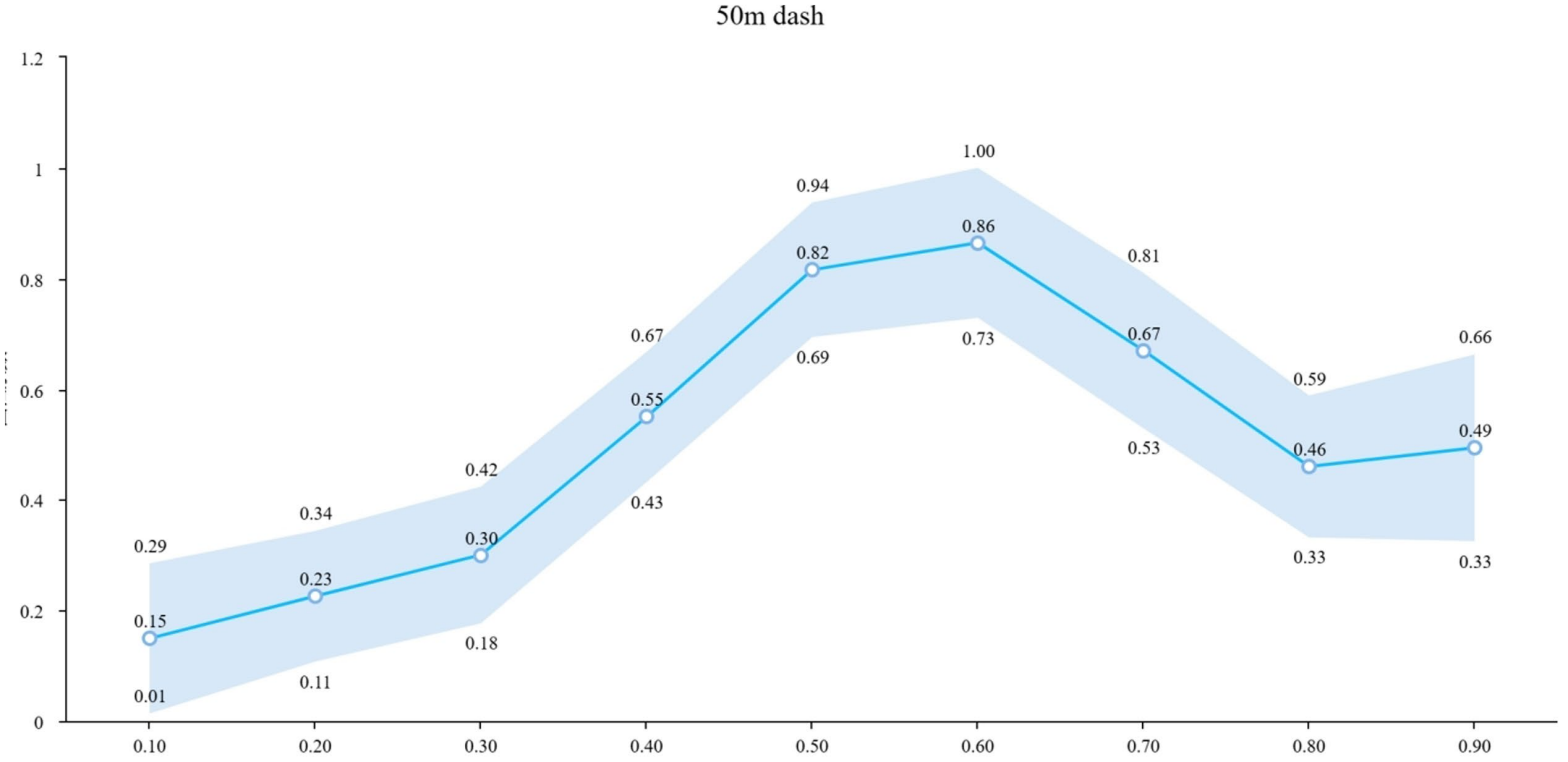
Quantile regression results for BMI and 50-meter sprint time. This figure shows the quantile-specific association between BMI (kg/m^2^) and 50-meter sprint time. The y-axis represents the BMI regression coefficient (β) for 50-meter sprint time, and the x-axis represents the quantile (τ) from 0.10 to 0.90. The shaded area represents the 95% confidence interval. Models were adjusted for gender, grade, and ethnicity.

**Figure 3:**
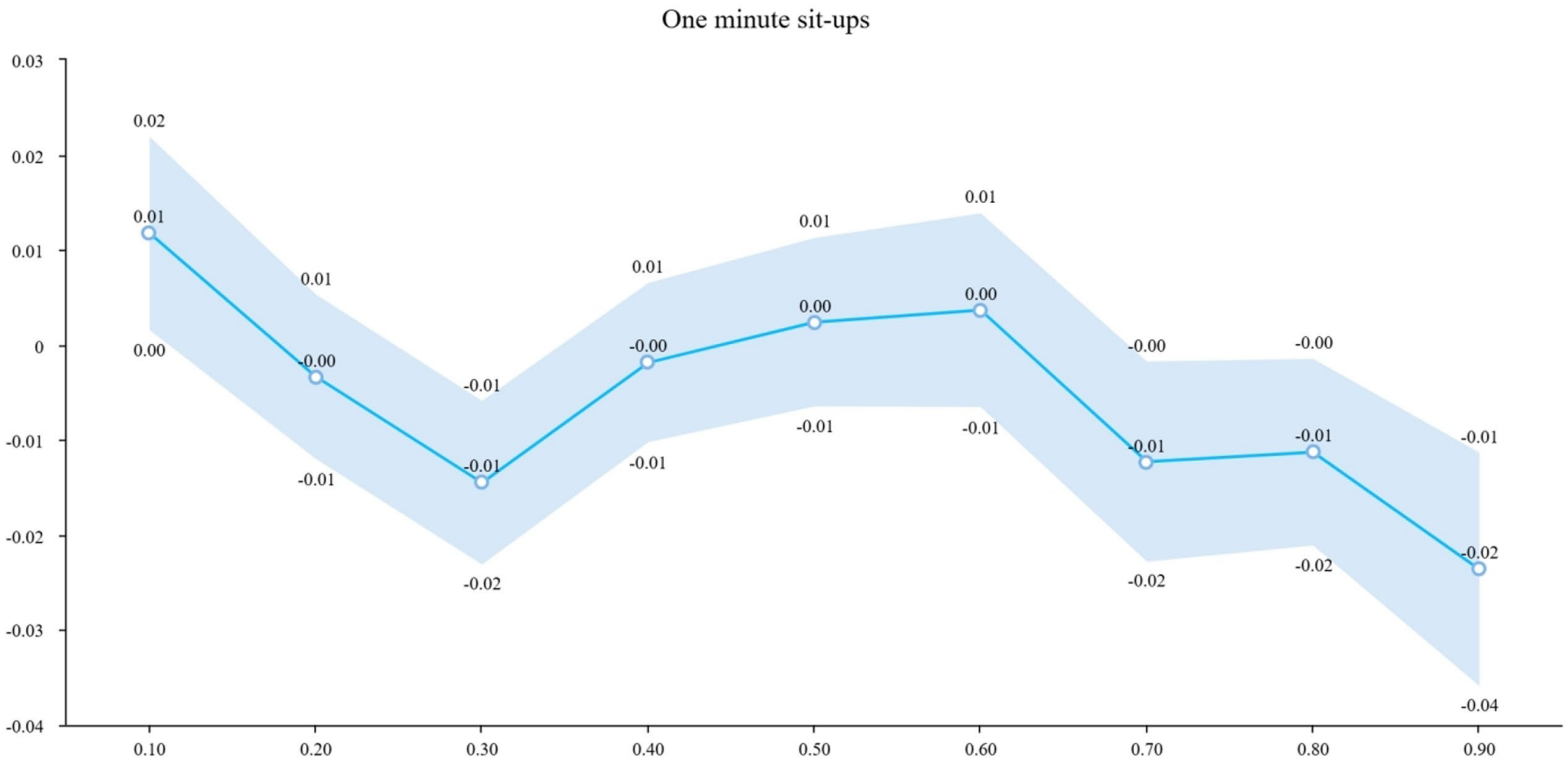
Quantile regression results for BMI and 1-min sit-ups. This figure shows the quantile-specific association between BMI (kg/m^2^) and 1-min sit-ups. The y-axis represents the BMI regression coefficient (*β*) for 1-min sit-ups, and the x-axis represents the quantile (τ) from 0.10 to 0.90. The shaded area represents the 95% confidence interval. Models were adjusted for gender, grade, and ethnicity.

**Figure 4:**
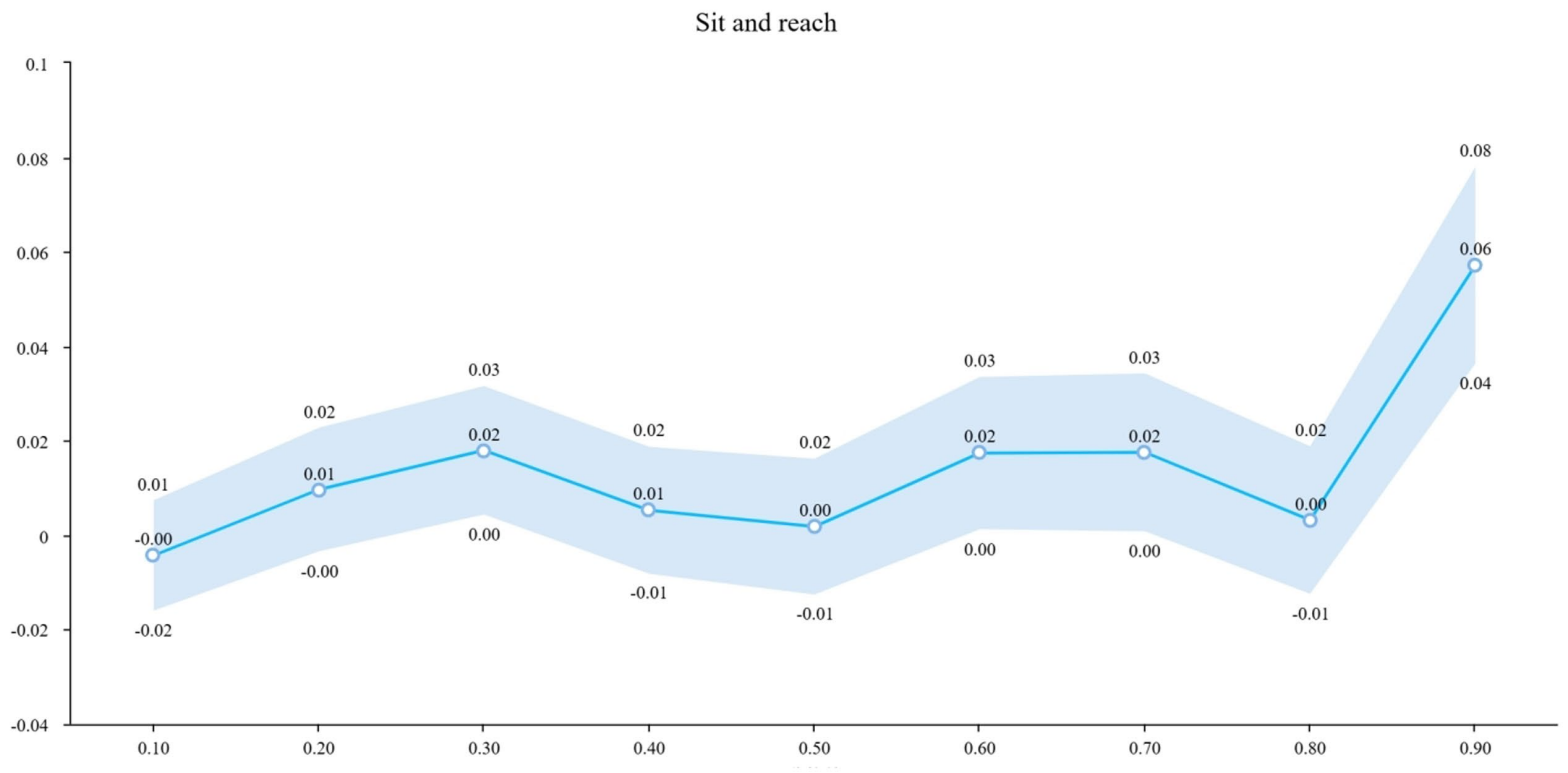
Quantile regression results for BMI and sit-and-reach performance. This figure shows the quantile-specific association between BMI (kg/m^2^) and sit-and-reach performance. The y-axis represents the BMI regression coefficient (β) for sit-and-reach distance, and the x-axis represents the quantile (τ) from 0.10 to 0.90. The shaded area represents the 95% confidence interval. Models were adjusted for gender, grade, and ethnicity.

**Figure 5:**
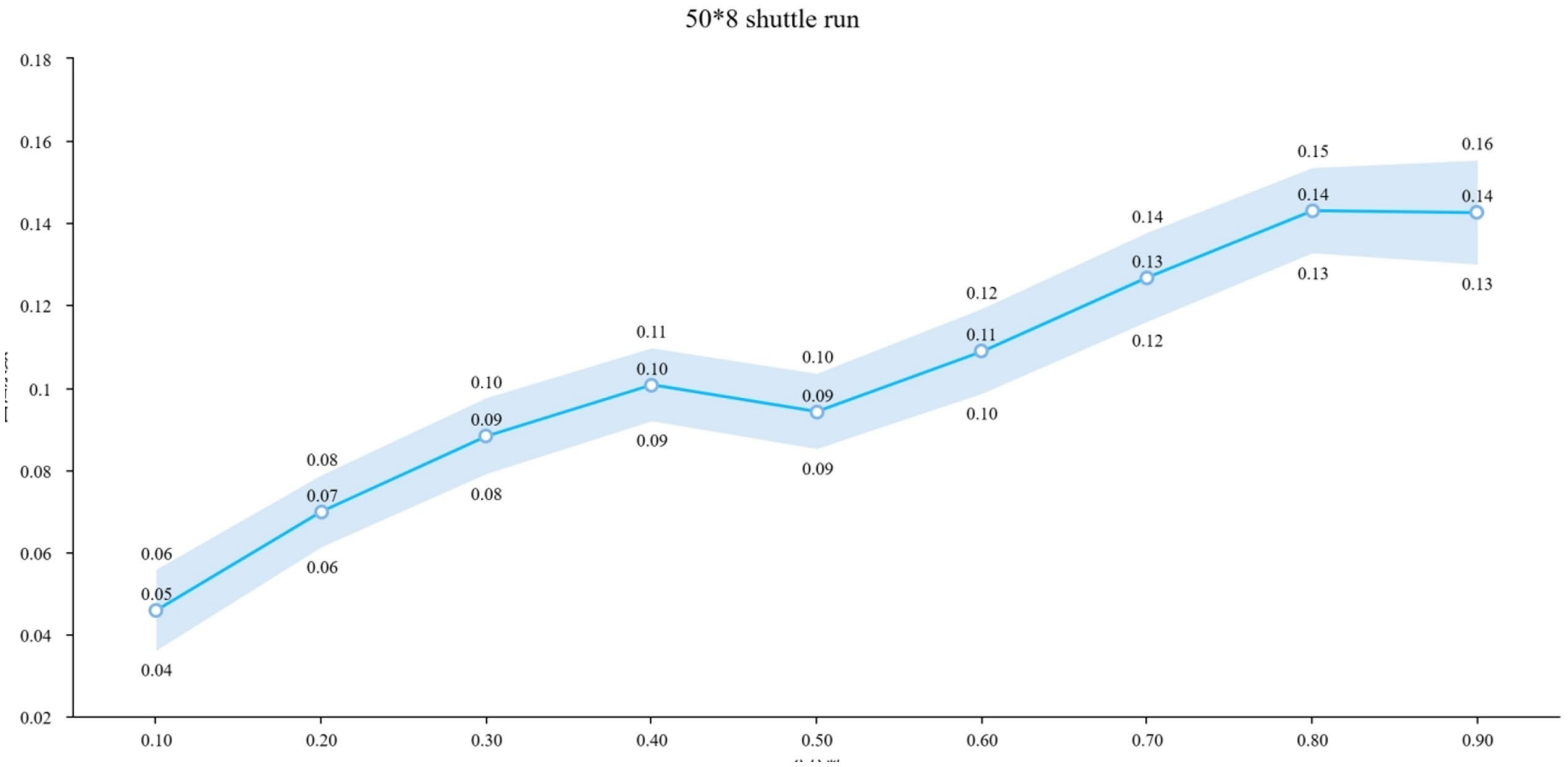
Quantile regression results for BMI and 50 × 8-meter shuttle run time. This figure shows the quantile-specific association between BMI (kg/m^2^) and 50 × 8-meter shuttle run time. The y-axis represents the BMI regression coefficient (β) for 50 × 8-meter shuttle run time, and the x-axis represents the quantile (τ) from 0.10 to 0.90. The shaded area represents the 95% confidence interval. Models were adjusted for gender, grade, and ethnicity.

**Figure 6:**
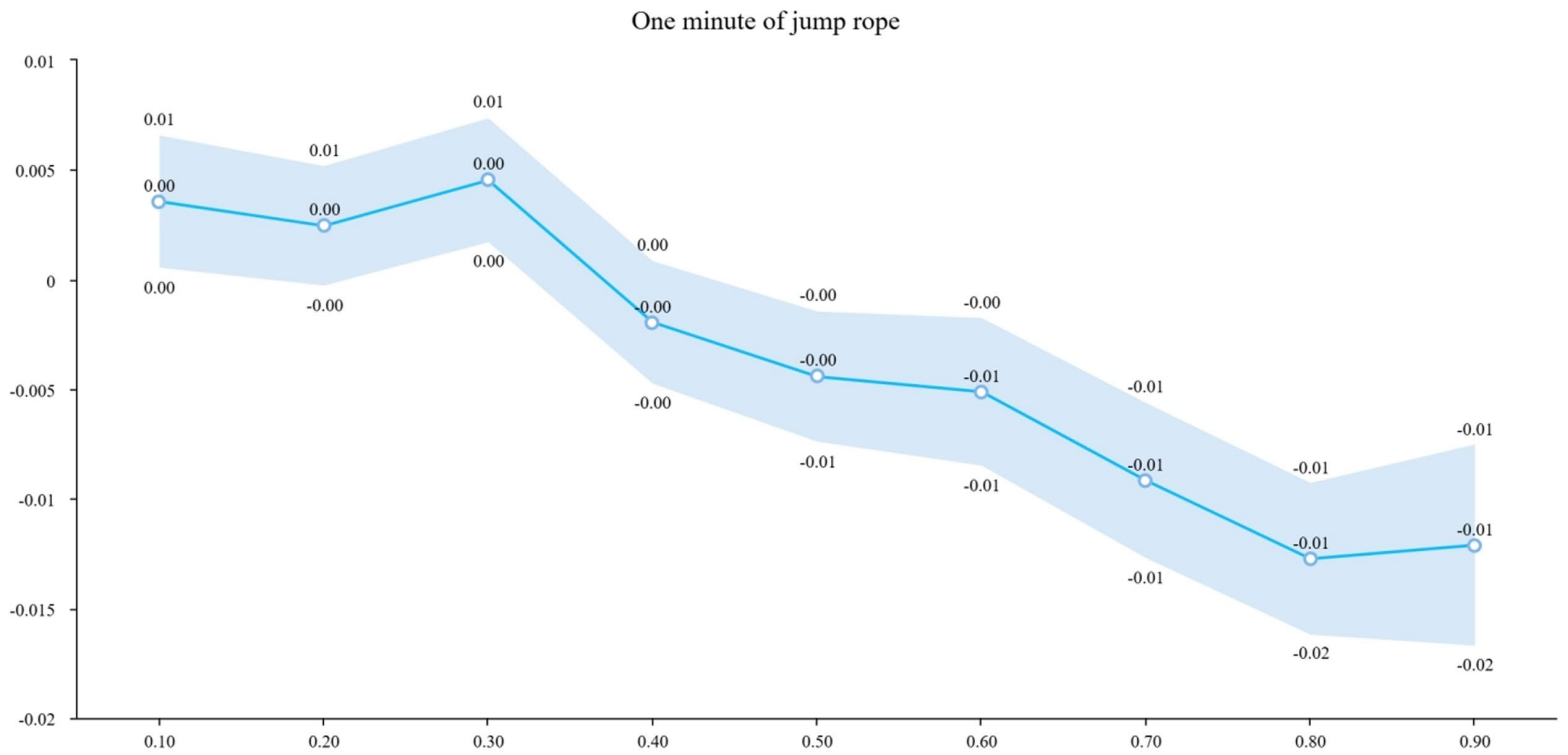
Quantile regression results for BMI and 1-min rope skipping. This figure shows the quantile-specific association between BMI (kg/m^2^) and 1-min rope skipping. The y-axis represents the BMI regression coefficient (β) for 1-min rope skipping, and the x-axis represents the quantile (τ) from 0.10 to 0.90. The shaded area represents the 95% confidence interval. Models were adjusted for gender, grade, and ethnicity.

Table 2 presents the quantile regression results in a standardized format. For each physical fitness outcome, the table reports the BMI coefficient, standard error, 95% confidence interval, and *p* value across all prespecified quantiles from 0.10 to 0.90. All models were adjusted for gender, grade, and ethnicity. To improve transparency and ensure consistency with Figures 1–6, all prespecified quantile estimates are shown in Table 2 regardless of statistical significance.

**Table 2:** Quantile regression coefficients for the association between BMI and physical fitness outcomes across quantiles.

Outcome	Quantile	Beta	SE	95% CI	*p* value
Lung capacity	0.1	0.002	0.001	0.001 to 0.002	<0.001
	0.2	0.002	0.001	0.002 to 0.002	<0.001
	0.3	0.002	0.002	0.002 to 0.003	<0.001
	0.4	0.003	0.001	0.002 to 0.003	<0.001
	0.5	0.003	0.001	0.002 to 0.003	<0.001
	0.6	0.003	0.001	0.002 to 0.003	<0.001
	0.7	0.003	0.001	0.003 to 0.003	<0.001
	0.8	0.003	0.002	0.003 to 0.003	<0.001
	0.9	0.004	0.001	0.003 to 0.004	<0.001
50-meter sprint time	0.1	0.149	0.069	0.014 to 0.285	0.031
	0.2	0.226	0.063	0.108 to 0.344	<0.001
	0.3	0.301	0.063	0.177 to 0.424	<0.001
	0.4	0.553	0.060	0.432 to 0.668	<0.001
	0.5	0.816	0.062	0.694 to 0.937	<0.001
	0.6	0.865	0.069	0.729 to 1.000	<0.001
	0.7	0.671	0.072	0.529 to 0.810	<0.001
	0.8	0.462	0.065	0.332 to 0.589	<0.001
	0.9	0.494	0.086	0.325 to 0.663	<0.001
Sit-and-reach	0.1	−0.002	0.008	−0.017 to 0.013	0.794
	0.2	0.006	0.006	−0.005 to 0.018	0.273
	0.3	0.018	0.007	0.004 to 0.032	0.009
	0.4	0.006	0.007	−0.007 to 0.019	0.382
	0.5	0.003	0.006	−0.009 to 0.015	0.624
	0.6	0.017	0.008	0.001 to 0.034	0.034
	0.7	0.018	0.009	0.001 to 0.034	0.039
	0.8	0.004	0.008	−0.012 to 0.020	0.617
	0.9	0.057	0.011	0.036 to 0.078	<0.001
1-min sit-ups	0.1	0.012	0.005	0.002 to 0.022	0.024
	0.2	−0.002	0.005	−0.012 to 0.008	0.697
	0.3	−0.014	0.004	−0.023 to −0.006	0.001
	0.4	−0.001	0.006	−0.012 to 0.010	0.859
	0.5	0.003	0.006	−0.009 to 0.015	0.624
	0.6	0.004	0.006	−0.008 to 0.016	0.513
	0.7	−0.012	0.005	−0.023 to −0.002	0.022
	0.8	−0.011	0.005	−0.021 to −0.001	0.024
	0.9	−0.024	0.006	−0.036 to −0.011	<0.001
1-min rope skipping	0.1	0.004	0.002	0.001 to 0.007	0.021
	0.2	0.003	0.002	−0.001 to 0.007	0.141
	0.3	0.005	0.001	0.002 to 0.007	0.002
	0.4	−0.002	0.002	−0.005 to 0.002	0.335
	0.5	−0.004	0.002	−0.007 to −0.001	0.003
	0.6	−0.005	0.002	−0.008 to −0.002	0.003
	0.7	−0.009	0.002	−0.013 to −0.006	<0.001
	0.8	−0.013	0.002	−0.016 to −0.009	<0.001
	0.9	−0.012	0.002	−0.017 to −0.008	<0.001
50 × 8-meter shuttle run time	0.1	0.046	0.005	0.036 to 0.056	<0.001
	0.2	0.072	0.004	0.061 to 0.079	<0.001
	0.3	0.088	0.005	0.079 to 0.097	<0.001
	0.4	0.101	0.005	0.092 to 0.110	<0.001
	0.5	0.094	0.005	0.085 to 0.103	<0.001
	0.6	0.109	0.005	0.098 to 0.119	<0.001
	0.7	0.127	0.006	0.116 to 0.138	<0.001
	0.8	0.143	0.005	0.133 to 0.153	<0.001
	0.9	0.143	0.006	0.130 to 0.155	<0.001

β indicates the estimated coefficient for BMI from quantile regression. SE indicates standard error. CI indicates confidence interval. Quantiles refer to the conditional distribution of each physical fitness outcome. All models were adjusted for gender, grade, and ethnicity. Positive coefficients for 50-meter sprint time and 50 × 8-meter shuttle run time indicate longer completion time and therefore poorer performance. All prespecified quantiles from 0.10 to 0.90 are reported regardless of statistical significance. Because the analyses were conducted on the final analytic sample without full design-based variance adjustment for the school-based cluster sampling design, the standard errors, confidence intervals, and *p* values should be interpreted with caution.

BMI was positively associated with lung capacity across all prespecified quantiles, with the coefficient increasing from 0.002 at the 0.10 quantile to 0.004 at the 0.90 quantile. For the 50-meter sprint, BMI was positively associated with sprint time across all prespecified quantiles, indicating that higher BMI was associated with longer sprint time and therefore poorer sprint performance. The coefficient increased from 0.149 at the 0.10 quantile to 0.865 at the 0.60 quantile and then decreased slightly at higher quantiles.

For sit-and-reach, BMI coefficients were estimated at all prespecified quantiles. Statistically significant positive associations were observed at selected quantiles, specifically at the 0.30, 0.60, 0.70, and 0.90 quantiles, whereas the associations at the 0.10, 0.20, 0.40, 0.50, and 0.80 quantiles were not statistically significant. This suggests that the association between BMI and flexibility was not uniform across the outcome distribution. For 1-min sit-ups, the association changed across quantiles. BMI was positively associated with sit-ups at the 0.10 quantile, while significant negative associations were observed at the 0.30 and 0.70–0.90 quantiles. The coefficients at the 0.20, 0.40, 0.50, and 0.60 quantiles were not statistically significant.

For 1-min rope skipping, BMI showed significant positive associations at the 0.10 and 0.30 quantiles, while significant negative associations were observed from the 0.50 to 0.90 quantiles. The associations at the 0.20 and 0.40 quantiles were not statistically significant. This pattern indicates that higher BMI was associated with fewer rope-skipping counts mainly among students in the middle-to-higher parts of the performance distribution. For the 50 × 8-meter shuttle run, BMI was positively associated with shuttle-run time across all prespecified quantiles, with coefficients increasing from 0.046 at the 0.10 quantile to 0.143 at the 0.80 and 0.90 quantiles, indicating poorer shuttle-run performance with higher BMI.

Overall, Table 2 shows that the BMI–fitness association differed substantially across physical fitness outcomes and quantiles. BMI showed consistently positive associations with lung capacity, 50-meter sprint time, and 50 × 8-meter shuttle run time across the prespecified quantiles, whereas the associations with sit-and-reach, 1-min sit-ups, and 1-min rope skipping varied across the outcome distribution. These results support the presence of distributional heterogeneity and indicate that the association between BMI and physical fitness cannot be fully characterized by mean-based estimates alone. These findings describe associations within the final analytic sample and should not be interpreted as population-weighted or causal estimates.

## 4 Discussion

This study used quantile regression to examine the association between BMI and physical fitness among primary school students aged 9–12 years in Zhejiang Province. The results showed clear quantile-specific heterogeneity in both the strength and, for some outcomes, the direction of the BMI–fitness associations across the conditional outcome distributions. These findings extend the traditional mean-based perspective (20, 21) by showing that BMI–fitness associations were not uniform across different parts of the physical performance distribution (4, 6, 22).

Quantile regression enabled examination of BMI–fitness associations beyond average effects by identifying how these associations differed across the physical performance distribution (20, 21, 23). This approach was appropriate for assessing quantile-specific heterogeneity and may help identify student subgroups that could benefit from more targeted school-based health management strategies (11, 24).

### 4.1 Findings

The findings showed clear heterogeneity in the association between BMI and physical fitness across outcome quantiles. For lung capacity, BMI was positively associated with the outcome across the prespecified quantiles, with somewhat larger coefficients in the higher quantiles (Figure 1). This is consistent with previous studies suggesting that children with higher BMI may also have greater lung capacity (22). One possible explanation is that body size may contribute to lung-function-related measures. However, this result should be interpreted cautiously because BMI cannot distinguish fat mass from lean mass or muscle mass. Therefore, the observed positive association should not be taken to mean that greater adiposity is beneficial for lung capacity; instead, it may partly reflect differences in overall body size or lean-mass-related characteristics. This pattern is broadly consistent with previous reports of nonlinear BMI–fitness relationships in adolescents (6, 22).

For the 50-meter sprint, higher BMI was associated with longer sprint time across all prespecified quantiles, with the largest coefficients observed around the middle quantiles, particularly at the 0.50 and 0.60 quantiles. This indicates poorer speed performance among children with higher BMI. Similar findings have been reported in earlier studies of school-aged populations (4, 24). A possible explanation is that excess body mass increases movement load and reduces running efficiency.

For flexibility, BMI coefficients were estimated across all prespecified quantiles. Significant positive associations between BMI and sit-and-reach performance were observed at selected quantiles, specifically at the 0.30 (*β* = 0.018), 0.60 (β = 0.017), 0.70 (β = 0.018), and 0.90 (β = 0.057) quantiles, with the largest coefficient observed at the 0.90 quantile. In contrast, the associations at the 0.10, 0.20, 0.40, 0.50, and 0.80 quantiles were not statistically significant. This pattern suggests that the association between BMI and sit-and-reach performance may vary across flexibility levels rather than remain constant throughout the distribution. Because BMI does not distinguish fat mass from lean mass, this finding should be interpreted cautiously. Nevertheless, it suggests that flexibility-related training may need to be tailored for students with different body composition profiles.

For 1-min sit-ups, the association with BMI was heterogeneous across the performance distribution, with a significant positive coefficient at the 0.10 quantile and significant negative coefficients at the 0.30 and 0.70–0.90 quantiles. The associations at the 0.20, 0.40, 0.50, and 0.60 quantiles were not statistically significant. This pattern suggests that the relationship between BMI and abdominal muscular endurance is not uniform across performance levels. One possible explanation is that, among children with better core endurance, the influence of BMI may differ because of variations in body composition, movement efficiency, or proximity to performance ceilings (11, 22).

Rope skipping showed weak and variable associations with BMI across the prespecified quantiles (Figure 6). Significant positive coefficients were observed at the 0.10 and 0.30 quantiles, whereas significant negative coefficients appeared from the 0.50 to 0.90 quantiles. The associations at the 0.20 and 0.40 quantiles were not statistically significant. This pattern may reflect the higher coordination demands of rope skipping. Children with higher BMI may experience greater difficulty in rhythm control, repeated jumping, and movement efficiency (7, 22).

For the 50 × 8-meter shuttle run, BMI was positively associated with shuttle-run time across all prespecified quantiles. Because a longer completion time indicates poorer performance, this finding suggests that higher BMI was consistently associated with lower shuttle-run performance (22). The coefficients tended to increase toward the higher quantiles, indicating that the association may be stronger among students with longer shuttle-run times.

Gender and grade were included as covariates in the models and may also be related to differences in physical fitness performance. In general, boys performed better in speed- and strength-related tasks, whereas girls tended to perform better in flexibility. These differences may reflect both biological factors and differences in activity preference during school age (25).

Grade-related patterns may be more complex. Although height, weight, and BMI generally increase with grade, speed-related performance may not improve consistently across all students. This finding suggests that school physical education should place equal emphasis on motor skill development and healthy weight management (18, 22, 24).

Because the sample included Han, She, Manchu, and Tujia students, ethnicity was included as a covariate in the models to account for potential differences related to ethnic background. In the present study, ethnicity was not used as a primary grouping variable for stratified analysis or BMI × ethnicity interaction analysis. Therefore, the present findings should not be interpreted as direct evidence of ethnic differences in BMI–fitness associations. The inclusion of multiple ethnic groups provides useful contextual information for understanding BMI–fitness associations among children in Zhejiang Province; however, ethnicity-specific inference requires further studies using ethnicity-stratified models, BMI × ethnicity interaction terms, and additional measures of habitual physical activity, dietary patterns, school sports participation, and local living environment (22, 25, 26).

Taken together, these findings indicate that BMI–fitness associations vary across performance levels and that mean-based models may obscure important distributional patterns. However, the results should be interpreted as sample-based associations rather than causal or population-weighted estimates.

Methodologically, several strengths should be noted. First, the study was based on a large school-based sample including multiple ethnic groups and used quantile regression to examine BMI–fitness associations across different parts of the physical performance distribution rather than only at the mean. Second, standardized school-based testing procedures improved the comparability and reproducibility of the physical fitness measurements. These features enhance the relevance of the findings for school health monitoring and physical education planning; however, the cross-sectional design, unmeasured confounding, and limits of sample-based inference should still be considered when interpreting the results.

### 4.2 Limitations

This study has several limitations. First, the cross-sectional design does not allow causal inference. Second, the sample was drawn from Zhejiang Province, which may limit the generalizability of the findings. Third, although students from four ethnic groups were included, the present analysis primarily treated ethnicity as an adjustment variable rather than conducting fully stratified cross-ethnic comparative models. Therefore, the ethnic dimension of the findings should be interpreted cautiously, and more in-depth ethnicity-specific analyses are needed in future research. Fourth, several potentially important confounders were not available, including daily physical activity level, dietary intake, sleep duration, screen time, socioeconomic status, and pubertal maturation status. These variables may influence both BMI and physical fitness and may partly explain the observed associations. Their absence may have resulted in residual confounding, and the direction and magnitude of some BMI–fitness associations could therefore be affected. Fifth, the analyses were conducted on the final analytic sample and did not fully incorporate design-based variance estimation for the school-based cluster sampling design; therefore, the standard errors, confidence intervals, and *p* values should be interpreted cautiously. Sixth, although quantile regression was appropriate for the distribution-focused research question, the present study did not formally compare it with alternative approaches for modeling overall nonlinear functional forms, such as spline-based methods or generalized additive models.

Future studies should use longitudinal designs to examine how BMI–fitness associations change over time. Broader sampling across different geographic and cultural settings would also improve external validity. In addition, future research could compare quantile regression with other flexible modeling strategies to better integrate distributional heterogeneity and overall nonlinear trend estimation. Such work would provide a more comprehensive basis for child health promotion.

### 4.3 Practical implications and educational insights

The findings have practical implications for school physical education and child health monitoring. First, schools may consider integrating BMI and physical fitness indicators into routine health surveillance to identify students who may require additional support, particularly those with higher BMI and poorer speed- or endurance-related performance. Second, physical education programs should be stratified according to both body characteristics and performance levels. For students with poorer speed-related or muscular-endurance performance, programs may emphasize healthy weight management, progressive aerobic activity, coordination training, and skill-specific exercises. Third, for students with relatively good flexibility but increasing BMI, dynamic movement training, core stability, and functional strength exercises may help prevent functional limitations.

In addition, culturally responsive physical education may be useful in multiethnic school settings (27). Incorporating regional and ethnic sports activities, where appropriate, may improve student engagement and promote regular participation in physical activity. However, because the present study was cross-sectional, these implications should be viewed as directions for school health promotion rather than evidence of intervention effectiveness.

## Data Availability

The datasets generated or analyzed during the current study are not publicly available but are available from the corresponding author upon reasonable request.
